# Adults Who Have Never Been Screened for Colorectal Cancer, Behavioral Risk Factor Surveillance System, 2012 and 2020

**DOI:** 10.5888/pcd19.220001

**Published:** 2022-04-21

**Authors:** Lisa C. Richardson, Jessica B. King, Cheryll C. Thomas, Thomas B. Richards, Nicole F. Dowling, Sallyann Coleman King

**Affiliations:** 1 Division of Cancer Prevention and Control, Centers for Disease Control and Prevention, Atlanta, Georgia

**Figure:**
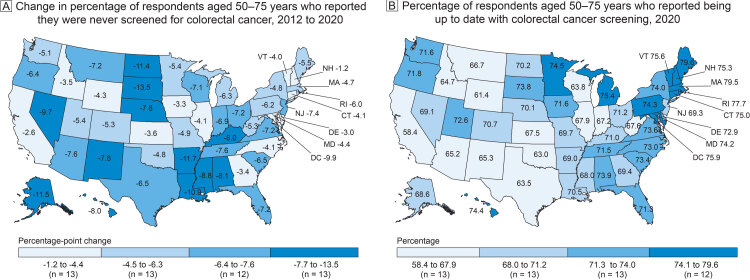
Colorectal cancer screening among US adults aged 50–75 years, Behavioral Risk Factor Surveillance System, 2012 and 2020. A, Change in percentage of US adults aged 50–75 years who reported they were never screened for colorectal cancer, 2012 to 2020. The overall decrease in never screened in the US was −5.8 percentage points. B, Percentage of US adults aged 50–75 years who reported being up to date with colorectal cancer screening in 2020. The percentage up to date in the US overall was 69.4%. Percentages were age-standardized to the 2000 US standard million population. Data source: Centers for Disease Control and Prevention, Behavioral Risk Factor Surveillance System (1,2).

**Table:** 

State	Map A. Percentage of adults aged 50–75 years who were never screened in 2020 minus the percentage never screened in 2012	Map B. Percentage of adults aged 50–75 years who reported being up to date with colorectal cancer screening in 2020
Alabama	−8.1	73.9
Alaska	−11.5	68.6
Arizona	−7.6	65.2
Arkansas	−11.7	69.0
California	−2.6	58.4
Colorado	−5.3	70.7
Connecticut	−4.1	75.0
Delaware	−3.0	72.9
District of Columbia	−9.9	75.9
Florida	−7.2	71.3
Georgia	−3.4	69.4
Hawaii	−8.0	74.4
Idaho	−3.5	64.7
Illinois	−4.1	67.9
Indiana	−6.9	67.2
Iowa	−3.3	71.6
Kansas	−3.6	67.5
Kentucky	−8.0	71.0
Louisiana	−10.9	70.5
Maine	−5.5	79.6
Maryland	−4.4	74.2
Massachusetts	−4.7	79.5
Michigan	−6.3	75.4
Minnesota	−5.4	74.5
Mississippi	−8.8	68.0
Missouri	−4.9	69.7
Montana	−7.2	66.7
Nebraska	−7.8	70.1
Nevada	−9.7	69.1
New Hampshire	−1.2	75.3
New Jersey	−7.4	69.3
New Mexico	−7.8	65.3
New York	−4.8	74.0
North Carolina	−4.1	73.0
North Dakota	−11.4	70.2
Ohio	−7.2	71.2
Oklahoma	−4.8	63.0
Oregon	−6.4	71.8
Pennsylvania	−6.2	74.3
Rhode Island	−6.0	77.7
South Carolina	−6.5	73.4
South Dakota	−13.5	73.8
Tennessee	−7.6	71.5
Texas	−6.5	63.5
Utah	−5.4	72.6
Vermont	−4.0	75.6
Virginia	−7.2	73.6
Washington	−5.1	71.6
West Virginia	−5.3	67.6
Wisconsin	−7.1	63.8
Wyoming	−4.3	61.4

## Background

In 2018, colorectal cancer (CRC) was the second most diagnosed cancer and the second leading cause of cancer death among cancers that affect both men and women (3). Screening for CRC can lead to fewer cases of cancer through the removal of polyps before they become cancer, the detection of cancers at their earliest stages, and the prevention of cancer deaths (4).

Studies from the UK of screening by sigmoidoscopy and from the US of screening by colonoscopy showed that even 1-time or infrequent screening has long-term benefits (5,6). Another study showed that 83% of people who were not up to date with CRC screening had never been screened and outlined multiple barriers to getting tested (7).

We measured the change in prevalence of adults who reported no CRC screening from 2012 to 2020. We also used data on the use of CRC screening tests in 2020 to update a previous report on up-to-date screening (8).

## Data and Methods

The Behavioral Risk Factor Surveillance System (BRFSS) is an annual, state-based, random-digit–dialed landline and cell phone survey of the civilian, noninstitutionalized US adult population aged 18 years or older. BRFSS collects information on demographic characteristics, health risk behaviors, preventive health practices, and health care access. We retrieved data on CRC screening from the 2012 and 2020 BRFSS (1,2). For consistency over time, we limited our analysis to respondents aged 50 to 75 years and applied the 2008 US Preventive Services Task Force (USPSTF) recommendations (9). We defined “up to date” as one of the following: 1) a home stool blood test (fecal occult blood test [FOBT] or fecal immunochemical test [FIT]) within 1 year, 2) sigmoidoscopy within 5 years with FOBT or FIT within 3 years, or 3) colonoscopy within 10 years. We analyzed the prevalence of respondents who responded yes when asked if they had ever had one of these tests and if yes, when they had the test. We defined “never screened” as respondents who answered no to being screened and respondents who had been screened but were not up to date per USPSTF 2008 recommendations. We excluded respondents who declined to answer or who reported “don’t know” or “not sure.” We used SAS-callable SUDAAN statistical software, version 9.4 (RTI International) for analysis. Results were age-standardized to the 2000 US standard million population to facilitate comparison with the Healthy People 2020 objective of 70.5% screened for CRC (10). We used ArcGIS Desktop version 10.8.1 (Esri) to create maps to show the absolute change in the percentage never screened between 2012 and 2020 and the percentage up to date in 2020. We used a 2-tailed Spearman rank correlation test to compare 1) the proportion of respondents by state reporting no CRC screening in 2012 with 2) the absolute difference by state in the proportion reporting no CRC screening in 2020 versus the proportion reporting no CRC screening in 2012.

## Highlights

The percentage of US adults never screened for CRC decreased from 27.4% in 2012 to 21.6% in 2020, a 5.8 percentage-point reduction representing 3,917,775 fewer people screened in 2012 than in 2020. Decreases ranged from 1.2 percentage points (New Hampshire) to 13.5 percentage points (South Dakota). Decreases were 8.0 percentage points or more in 10 states and the District of Columbia (Map A). The percentage of adults never screened was higher in the northern Great Plains and the Deep South. States with the largest improvements in the proportion never screened were those with the largest proportion never screened in 2012 (Figure).

**Figure:**
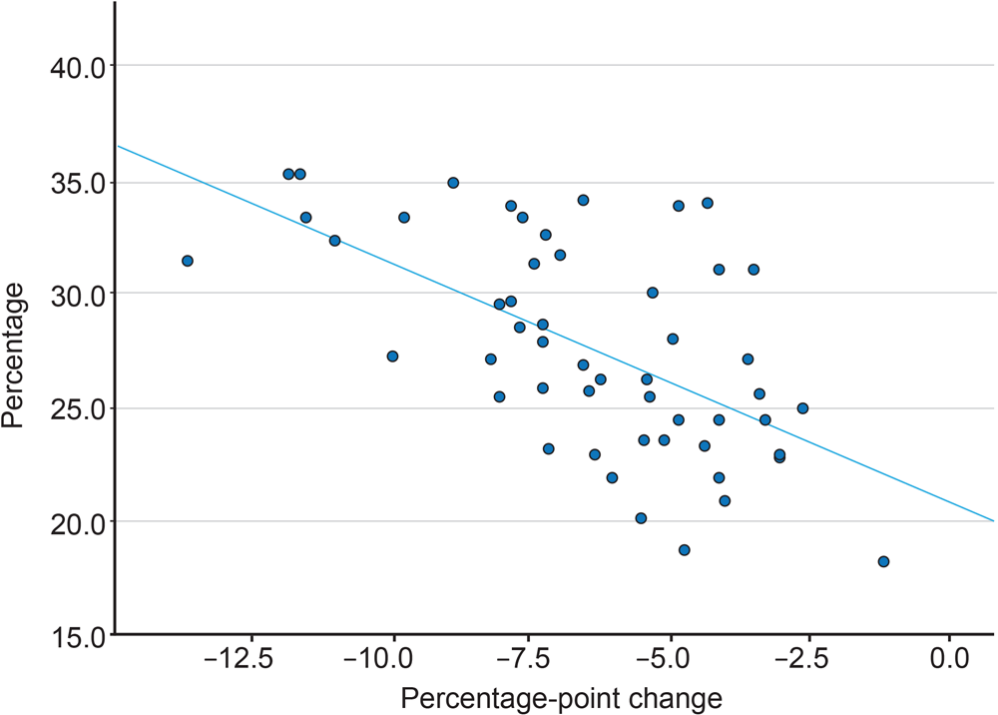
Correlation between 1) the percentage never screened for colorectal cancer in 2012 and 2) the absolute difference in the percentage never screened in 2020 minus the percentage never screened in 2012, by state. Each dot represents a state or the District of Columbia. Spearman *r* = −0.58; *P* = .01 (2-tailed). Data source: Centers for Disease Control and Prevention, Behavioral Risk Factor Surveillance System (1,2).

The percentage of adults aged 50 to 75 years who reported being up to date with CRC screening in 2020 was 69.4%, representing 62.3 million age-eligible adults, ranging from 58.4% in California to 79.6% in Maine (Map B). The percentage of up-to-date screening tended to be higher in New England. Twenty-two states did not meet the Healthy People 2020 objective of 70.5% screened for CRC.

## Action

The proportion of US adults never screened for CRC decreased from 2012 to 2020 in all states and the District of Columbia. The greatest increases were in states with the highest prevalence of never screened in 2012. Even with differences in the definition of never being screened, we found improvements in the percentage screened from the approximately 29% of respondents aged 50 to 75 years never screened according to 2010 BRFSS data (5). Nevertheless, CRC screening prevalence remains lower than desired. Given the challenges of the ongoing COVID-19 pandemic, the new Healthy People 2030 target of 74.4% will likely be hard to reach.

USPSTF recommendations were updated in 2016 to include more types of screening tests (2). In 2020 for the first time, BRFSS included questions on stool DNA testing and computerized tomographic colonography (11). When we included all 5 CRC testing methods, 71.6% of respondents aged 50 to 75 years reported being up to date with CRC screening in 2020.

The National Colorectal Cancer Roundtable, in collaboration with the Centers for Disease Control and Prevention (CDC), renewed a call to action to increase CRC screening to 80% (12). This call to action must address persons aged 45 to 49 years who are now eligible for screening (2) in addition to persons aged 50 to 75 years who have never been screened. The latter group comprises most people who are not up to date.

Financial and nonfinancial barriers might explain differences in screening by state. Fedewa and colleagues noted that states that expanded Medicaid soonest after the Affordable Care Act was enacted in 2010 had the largest increases in CRC screening (13). We found a correlation between the states with the largest proportion of people never screened and improvements in screening among people never screened. States with the smallest decreases in people never screened were concentrated in the South, where Medicaid expansion still has not occurred. In contrast, South Dakota has not expanded Medicaid, but it had the largest improvement (−13.5 percentage points) among people never screened. One possible explanation is that South Dakota has been a part of CDC’s Colorectal Cancer Control Program for over a decade. This program focuses on using evidence-based strategies to increase CRC screening (14). In a study that examined reasons for not being screened, people with low educational attainment, no health insurance, and no usual source of care had the highest prevalence of never being screened (5).

Nonfinancial factors also affect CRC screening. Jones and colleagues published a report of patient-reported barriers to CRC screening in 2010 (15). In their mixed-methods study, which included African American people and people with low income, barriers identified were lack of understanding about what to do when being screened and what screening involved, lack of motivation to get tested because of reservations about getting the test, and not having the means to pay for initial testing and possible follow-up testing. No similar studies have been conducted among people who reported never being screened for CRC. Reducing these barriers will require developing educational resources designed to meet the needs of people who experience these barriers

Our study has several limitations. First, CRC screening prevalence may be underestimated or overestimated because of recall bias. Second, we were unable to differentiate between a screening test and a diagnostic test, and respondents may not have been able to differentiate between types of stool tests and endoscopies. Third, social desirability bias could have affected responses to survey questions. Fourth, our analysis did not account for any sampling error. Fifth, the response rate for BRFSS was about 45%, and some respondents did not answer all the questions. Lastly, National Health Interview Survey data are used to determine Healthy People national objectives, whereas BRFSS data are used to measure state-level progress toward improving health behaviors that affect chronic diseases (16). Estimates from BRFSS tend to be higher than estimates from the National Health Interview Survey, possibly because of the survey methods (17).

If we are to reach the Healthy People 2030 objective of 74.4% of the population screened for CRC or the goal of 80% screened in every community (12), we should intensify outreach to people who have never been screened, because most of those not up to date have never been screened (5). During the COVID-19 pandemic, the backlog in CRC screening has grown to nearly 4 million people (18). We have a lot of work ahead of us. The President’s Cancer Panel released new recommendations in early 2022 that will inform this work (19).

## References

[1] Centers for Disease Control and Prevention. BRFSS 2012 survey data and documentation. https://www.cdc.gov/brfss/annual_data/annual_2012.html. Accessed December 5, 2021.

[2] Centers for Disease Control and Prevention. 2020 BRFSS survey data and documentation. https://www.cdc.gov/brfss/annual_data/annual_2020.html. Accessed December 5, 2021.

[3] Centers for Disease Control and Prevention. United States cancer statistics: data visualizations. June 2021. https://www.cdc.gov/cancer/dataviz. Accessed December 9, 2021.

[4] US Preventive Services Task Force. Final recommendation statement. Colorectal cancer: screening. May 18, 2021. https://www.uspreventiveservicestaskforce.org/uspstf/recommendation/colorectal-cancer-screening. Accessed December 5, 2021.

[5] Atkin W , Wooldrage K , Parkin DM , Kralj-Hans I , MacRae E , Shah U , Long term effects of once-only flexible sigmoidoscopy screening after 17 years of follow-up: the UK Flexible Sigmoidoscopy Screening randomised controlled trial. Lancet 2017;389(10076):1299–311. 10.1016/S0140-6736(17)30396-3 28236467 PMC6168937

[6] Lee JK , Jensen CD , Levin TR , Zauber AG , Schottinger JE , Quinn VP , Long-term risk of colorectal cancer and related deaths after a colonoscopy with normal findings. JAMA Intern Med 2019;179(2):153–60. 10.1001/jamainternmed.2018.5565 30556824 PMC6439662

[7] Stanley SL , King JB , Thomas CC , Richardson LC . Factors associated with never being screened for colorectal cancer. J Community Health 2013;38(1):31–9. 10.1007/s10900-012-9600-x 22875234 PMC12176307

[8] Joseph DA , King JB , Richards TB , Thomas CC , Richardson LC . Use of colorectal cancer screening tests by state. Prev Chronic Dis 2018;15:E80. 10.5888/pcd15.170535 29908051 PMC6016405

[9] US Preventive Services Task Force. Final recommendation statement. Colorectal cancer: screening. October 15, 2008. https://www.uspreventiveservicestaskforce.org/uspstf/recommendation/colorectal-cancer-screening-2008. Accessed December 5, 2021.

[10] Office of Disease Prevention and Health Promotion. Healthy People topics & objectives. Cancer. https://www.healthypeople.gov/2020/topics-objectives/topic/cancer. Accessed November 13, 2021.

[11] Centers for Disease Control and Prevention. Behavioral Risk Factor Surveillance System. Overview, BRFSS 2020. https://www.cdc.gov/brfss/annual_data/2020/pdf/overview-2020-508.pdf. Accessed July 7, 2021.

[12] National Colorectal Cancer Roundtable. 80% in Every Community. Achieving 80% colorectal cancer screening rates in every community. https://nccrt.org/80-in-every-community. Accessed December 5, 2021.

[13] Fedewa SA , Yabroff KR , Smith RA , Goding Sauer A , Han X , Jemal A . Changes in breast and colorectal cancer screening after Medicaid expansion under the Affordable Care Act. Am J Prev Med 2019;57(1):3–12. 10.1016/j.amepre.2019.02.015 31128952

[14] Centers for Disease Control and Prevention. Colorectal Cancer Control Program (CRCCP). https://www.cdc.gov/cancer/crccp/index.htm. Accessed September 2, 2021.

[15] Jones RM , Devers KJ , Kuzel AJ , Woolf SH . Patient-reported barriers to colorectal cancer screening: a mixed-methods analysis. Am J Prev Med 2010;38(5):508–16. 10.1016/j.amepre.2010.01.021 20409499 PMC2946825

[16] Centers for Disease Control and Prevention. Chronic disease indicators. https://nccd.cdc.gov/cdi. Accessed December 14, 2021.

[17] Sauer AG , Liu B , Siegel RL , Jemal A , Fedewa SA . Comparing cancer screening estimates: Behavioral Risk Factor Surveillance System and National Health Interview Survey. Prev Med 2018;106:94–100. 10.1016/j.ypmed.2017.10.019 29079098

[18] Chen RC , Haynes K , Du S , Barron J , Katz AJ . Association of cancer screening deficit in the United States with the COVID-19 pandemic. JAMA Oncol 2021;7(6):878–84. 10.1001/jamaoncol.2021.0884 33914015 PMC8085759

[19] President’s Cancer Panel. Closing gaps in cancer screening: connecting people, communities, and systems to improve equity and access. A report from the President’s Cancer Panel to the President of the United States. Bethesda (MD): President’s Cancer Panel; 2022.

